# Pulsatile Physiological Control of Blood Pump-Cardiovascular System Based on Feedforward Compensation

**DOI:** 10.3390/mi16060664

**Published:** 2025-05-31

**Authors:** Yanjun Bao, Teng Jing, Weimin Ru, Ling Zhou

**Affiliations:** Research Center of Fluid Machinery Engineering & Technology, Jiangsu University, Zhenjiang 212013, China

**Keywords:** Rotary Blood Pump, feed-forward compensation, pulsatility, physiological, cardiovascular system

## Abstract

Rotary Blood Pump (RBP) is a commonly used ventricular assist device. However, the constant speed operation of the blood pump leads to a reduction of blood flow pulsatility, which triggers a series of adverse reactions. In this paper, a pulsatile physiological control with feed-forward compensation (FFC) is designed to regulate the rotational speed in real time to accurately output pulsatile blood flow to address this problem. The coupled model of the Rotary Blood Pump and cardiovascular system (CVS) is established in the SIMULINK software as the research object. The designed pulsatile physiological control algorithm contains the feed-forward compensation-based pulsatile control and anti-reflux algorithm, switching the applicable algorithm based on the pump flow. When the flow rate is higher than the threshold, feed-forward compensation is introduced and combined with PI feedback control to improve the performance of pulsation tracking; when the flow rate is lower than the threshold, it is switched to the anti-reflux algorithm to gradually increase the pump speed. Simulation shows that the designed feed-forward compensation link reduces the tracking error of the pulsatile physiological control by 80%. In the case of a 50% sudden change of physiological parameters, it can track quickly and stably and avoid reflux. The pulsatile performance and ventricular unloading performance are better compared with no feed-forward compensation pulsation control as well as constant-speed control. An increase of 30 mmHg in aortic beat-to-beat differential pressure was achieved in the extracorporeal circulation experiments, which is important for the realization of pulsatile flow control of the Rotary Blood Pump.

## 1. Introduction

The Rotary Blood Pump (RBP) is widely used for the transition to heart transplantation or even permanent therapy in patients with severe heart failure due to its high stability, small size, and simple structure. By modulating to a reasonable rotational speed, the RBP can ensure the basic blood perfusion volume, but running at a constant speed in long-term assistance can cause related complications and adverse events [1,2]. The most important cause of these complications is the low shear stress on the endothelial cells of the vessel wall when blood flows through the vessel. By adjusting the rotational speed in real time so that the RBP outputs pulsatile blood flow, it can increase the pulsatility of the blood vessels, improve the shear stress, and maintain the normal function of the endothelial cells, and the pulsatile blood flow has the effect of dilating the small arteries and decreasing the vascular resistance, which reduces a series of clinical complications.

Currently, in the study of enhancement of blood flow pulsatility, Ising, M.S.et al. [3] evaluated the amplitude, phase, and frequency of three types of RBP tacho modulation for the enhancement of blood flow pulsatility, and analyzed the effects of asynchronous, synchronous, and synchronous counterpulsation of RBP on blood pulsatility and ventricular unloading. In vitro circulation experiments and animal experiments demonstrated that asynchronous modulation produced the greatest vascular pulsatility, whereas synchronous modulation reduced the most left ventricular work done. M. Arakawa et al. [4]. combined ECG monitoring to keep the RPM low in systole and high in diastole to increase blood flow pulsatility. In these studies, the setting of the RPM values lacked a physiological basis, and the modulation pattern of RPM was fixed and open-loop, with limited adaptability to hemodynamic parameters and cardiac load, which made it easy for adverse events such as regurgitation to occur [5]. This regurgitation not only reduces the efficiency of the RBP but also has adverse effects on the myocardium in the long term, such as interstitial cardiac fibrosis and decreased myocardial compliance [6]. Wang et al. [7]. defined regurgitation index (RI) based on the pump flow signal and determined the RI intervals of different regurgitation states through open-loop and fixed-speed control of the LVAD, which realized the detection and classification of regurgitation in the LVAD.

A better solution is to develop a closed-loop control strategy for the rotational speed by using the RBP rotational speed as the control variable and the hemodynamic index as the control target, forming a feedback loop. Huang et al. [8] used the mean arterial pressure and pulse pressure index extracted from the aortic pressure signal as the feedback signals, regulated the mean rotational speed and the rotational speed amplitude by two fuzzy controllers, respectively, and used Anti-Reflux Control to prevent the emergence of reflux in the RBP, which demonstrated the effectiveness of the algorithm under various physiological disturbances through numerical simulation and was able to increase the difference in pulse pressure up to 20 mmHg on a real RBP. Bakouri et al. [9] utilized the pump flow pulsatility index to determine the time-varying desired mean pump flow, used estimation to indirectly measure the pump flow, and validated the control strategy in a numerical model, which showed that this control system was able to adapt to changes in ventricular loading due to blood loss and exercise without the occurrence of ventricular pumping; however, this method relies on autologous ventricular systolic function and is only applicable to patients with a low degree of heart failure. Arndt’s team [10] innovatively extracted a gradient index of the rate of change of pulsation intensity with respect to rotational speed based on the axial thrust measured by the pump’s magnetic bearings, (defined as Gradient Pulsatility Index, GPI) and also designed a polar search control algorithm, which can satisfy a variety of control objectives through the cascade of GPI and PI, and the Animal experiments have been completed.

In order to improve the blood pulsatility of patients with end-stage heart failure and to adjust the RBP perfusion in real time according to the physiological activity level, this paper proposes a control strategy based on feed-forward compensation (FFC), using MATLAB 2023b software, to establish the RBP-cardiovascular coupling model. Based on this model and the hemodynamic characteristics of heart failure patients, a second-order feed-forward compensation (FFC) controller was designed, and the tracking performance of pulsatile aortic pressure under different control strategies and the overall auxiliary performance were comparatively analyzed using simulation methods and verified by experiments.

## 2. Models and Methods

### 2.1. Mathematical Model

In this study, a set-parameter mathematical model was developed to describe the kinetics of the CVS based on its anatomical characteristics and the parallel implantation form of the RBP. The CVS equivalent circuit model is constructed by comparing the blood flow with the circuit flow through the Navier-Stokes equations and characterizing the vascular resistance, compliance and blood inertia by using the resistance, capacitance and inductance equivalent circuit elements, as well as by introducing the left ventricular time-varying inverse capacitance function to describe the elastic chamber characteristics of the heart and diodes in series with resistors to simulate the function of the heart valves. The RBP model was established from the hydraulic performance test data, and the equivalent circuit model was further extended to the coupled system including the RBP. The state equations are established according to Kirchhoff’s law, and finally, the dynamic model of the whole system is constructed and verified in SIMULINK software.

#### 2.1.1. Vascular Network Model

It is assumed that the arterial vasculature is a cylindrical elastic tube and that the blood flows in a uniform unidirectional laminar flow through the vessel, and gravity is neglected. According to the Navier-Stokes equations [11], blood flow in a blood vessel has a similar relationship to current flow in an electric circuit. As shown in Equation (1).

(1) $$\left\{\begin{array}{l} -\frac{\partial P}{\partial z}=\frac{\rho }{\pi r^{2}}\cdot \frac{\partial Q}{\partial t}+\frac{8\mu }{\pi r^{4}}\cdot Q\\ -\frac{\partial Q}{\partial z}=\frac{3\pi r^{3}}{2Ed}\cdot \frac{\partial P}{\partial t} \end{array}\right.$$

**Figure 1:**
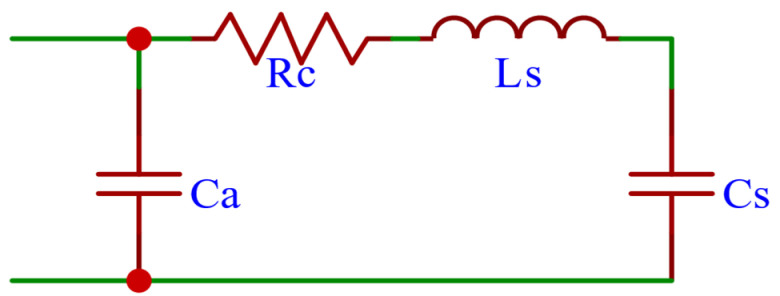
Equivalent circuit modeling of vascular networks [12].

Using electrical circuits with resistance equivalent to the resistance to flow of the vessel, capacitance equivalent to vessel compliance, and inductance equivalent to the inertia of the blood during flow, blood flow and blood pressure are characterized using currents and voltages, and the hemodynamic parameters and LRC electrical parameters are compared according to Equation (1) and the hemodynamic parameter calculations for a vessel of length l are expressed as Equation (2):

(2) $$\left\{\begin{array}{l} R=\frac{8\mu }{\pi r^{4}}\cdot l\\ L=\frac{\rho }{\pi r^{2}}\cdot l\\ C=\frac{3\pi r^{3}}{2Ed}\cdot l \end{array}\right.$$

where *P* is blood pressure, *Q* is blood flow, *ρ* is fluid density, *μ* is blood flow viscosity, *r* is vessel radius, *d* is wall thickness, and *E* is Young’s modulus. According to the anatomical structure and physiological properties of the cardiovascular system, As shown in Figure 1, the classical Windkessel four-element elastic lumen model can be obtained.

#### 2.1.2. Heart Model

The natural human heart contains atria, ventricles, and the heart valves in between, of which the left ventricle is an elastic chamber whose periodic contraction and diastole are the power sources of blood circulation. The ratio, *E*, of left ventricular pressure to volume is expressed as the time-varying inverse capacitance function, *E*(*t*). Its value varies with the systolic state [13]. The change in compliance of the left ventricle of an elastic chamber is as follows:

(3) $$C(t)=\frac{1}{E(t)}$$

(4) $$E(t)=(E_{\max}-E_{\min})\cdot E_{n}(t_{n})+E_{\min}$$

where *C*(*t*) is the left ventricular equivalent capacitance function, *E*_max_ is the left ventricular end-systolic compliance, when the ventricular active elasticity reaches its maximum, and *E_min_* is the left ventricular end-diastolic compliance. *E*_n_(*t*_n_) is the normalized function of *E*(*t*), and the specific expression is shown in the following Equation (13):

(5) $$\left\{\begin{array}{l} E_{n}(t_{n})=1.55\cdot \frac{{\left(t_{n}/0.7\right)}^{1.9}}{1+{\left(t_{n}/0.7\right)}^{1.9}}\cdot \frac{1}{1+{\left(t_{n}/1.17\right)}^{21.9}}\\ t_{n}=\frac{t}{t_{\max}}\\ t_{\max}=0.2+\frac{9}{HR} \end{array}\right.$$

where *t*_n_ is the normalized time; *t_max_* is left ventricular end-diastole; *HR* is heart rate.

After finding the time-varying inverse capacitance *E*(*t*), the pressure in the left ventricle *Plv*(*t*) can be calculated at different volumes:

(6) $$Plv(t)=E(t)\cdot [Vlv(t)-V_{0}]$$

#### 2.1.3. Heart Valve Model

Heart valves are similar to one-way valves, preventing the backflow of blood. The valve coefficient of regurgitation is a value between 0 and 1. The ideal valve has a coefficient of regurgitation of 0. The valve can be replaced by a diode in a circuit element in series with a resistor equivalent. The mathematical expression for the flow blocked by the valve is given in Equation (7). Where *ξ* is the flow rate through the valve.

(7) $$r(\xi )=\left\{\begin{array}{l} 0,if\xi \leq 0\\ \xi ,if\xi >0 \end{array}\right.$$

#### 2.1.4. RBP Model

**Figure 2:**
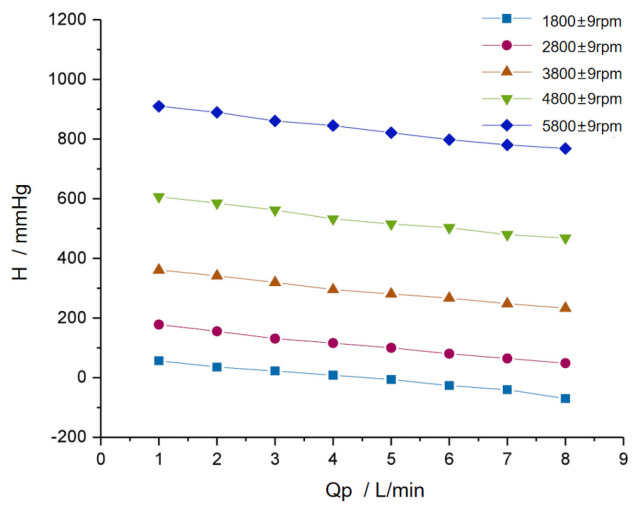
Hydraulic performance testing of the RBP.

The hydraulic performance test of the RBP (Figure 2) allows us to obtain the relationship between the pressure difference *H* between the RBP and the casing connecting the two ends of the RBP, as well as the flow rate *Qp* and the rotational speed *ω*, can be obtained in Equation (8).

(8) $$H=Rp\cdot Qp+Lp\cdot \frac{dQp}{dt}+\beta \cdot \omega^{2}$$

where *Rp* is the resistance coefficient of the pump and casing, *Lp* is the inertia coefficient of the pump and casing, which are equivalent to resistance and inductance, respectively, and *β*·*ω*^2^ is the pressure source determined by the rotational speed of the RBP, which can be equivalent to the voltage source *u*(*t*) with variable input.

#### 2.1.5. CVS-RBP Equivalent Circuit Model

**Figure 3:**
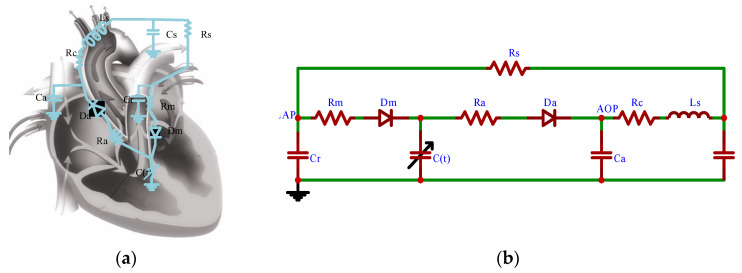
CVS schematic and Equivalent Circuit. (**a**) Cardiovascular Schematic. (**b**) Equivalent circuit diagram.

**Figure 4:**
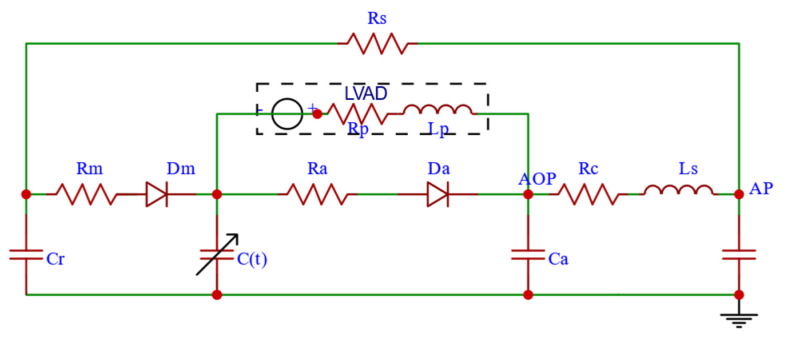
Equivalent circuit of CVS-RBP coupling model when implanted in parallel.

**Figure 5:**
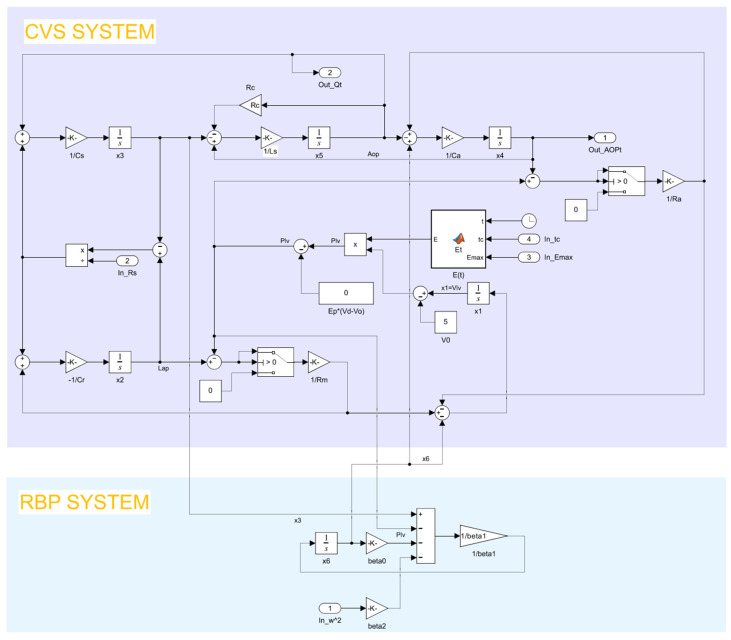
SIMULINK modeling of the CVS-RBP coupled system.

The CVS equivalent circuit model was established according to the equivalent circuit method as shown in Figure 3, where *Lap*, *Lvp*, *Aop*, *Ap*, *Vp*, and *Q* represent left atrial pressure, left ventricular pressure, aortic pressure, arterial pressure, venous pressure, and aortic flow. When the RBP is implanted in parallel, the coupled CVS-RBP circuit model is shown in Figure 4. According to Kirchhoff’s circuit law, the equation of state is established in Equation (9). The controlled object model is built in SIMULINK software as shown in Figure 5. The physiological significance of each parameter of the CVS equivalent circuit model is shown in Table 1. The state variables are shown in Table 2.

(9) $$\left\{\begin{array}{l} \frac{dx_{1}}{dt}=r[x_{2}-E(t)\cdot (x_{1}-V_{0})]/Rm-r[E(t)\cdot (x_{1}-V_{0})-x_{4}]/Ra\\ \frac{dx_{2}}{dt}=\left\{(x_{3}-x_{2})/Rs-r[x_{2}-E(t)\cdot (x_{1}-V_{0})]/Rm\right\}/Cr\\ \frac{dx_{3}}{dt}=[x_{5}-(x_{3}-x_{2})/Rs]/Cs\\ \frac{dx_{4}}{dt}=\left\{r[E(t)\cdot (x_{1}-V_{0})-x_{4}]/Ra-x_{5}\right\}/Ca+x_{6}\\ \frac{dx_{5}}{dt}=[x_{4}-x_{3}-Rc\cdot x_{5}]/Ls\\ \frac{dx_{6}}{dt}=[x_{4}-E(t)\cdot (x_{1}-V_{0})-Rp\cdot x_{6}-\beta \cdot \omega^{2}]/Lp \end{array}\right.$$

### 2.2. Control Strategies

The designed pulsatile physiological control algorithm consists of two parts: the FFC-based pulsatile control algorithm and the anti-reflux algorithm. It relies on the pump flow rate at the current moment to determine which control algorithm is enabled.

#### 2.2.1. Selection of Desired Aortic Pressure Signals

The desired aortic pressure signal not only serves as a reference trajectory that the physiological control system expects to achieve, but also serves as an input signal to the FFC channel to accelerate the system’s response to target changes. Therefore, the selection of the desired aortic pressure signal depends on the effectiveness of the whole control scheme. The regular waveforms of time-varying desired aortic pressure signals are square, triangular, and sinusoidal, in addition to different amplitudes, frequencies, and phase shifts that need to be taken into account. In this study, a multi-objective optimization framework was designed to achieve gentle tracking of the target pressure [14], along with convenient configuration of the input mean aortic pressure and pulse pressure. In this paper, a sinusoidal waveform is selected as the target waveform. The expression for the sinusoidal pulsating waveform is Equation (10).

In practice, healthcare professionals can customize the desired mean aortic pressure and pulse pressure for patients by manipulating *Aop*_0_ and *Aop*_a_. The value of *Aop*_0_ represents the mean value of the target aortic pressure, and *Aop*_a_ is the amplitude, and twice its value represents the target aortic pulse pressure. From the point of view of the most beneficial to the recovery of the failing heart, we select the synchronous coupling waveform [15] that is consistent with the frequency and phase of the HR to produce a pulsatile blood flow that is more in line with the physiological needs. At this point, the pulsation modulation frequency coincides with the natural heart beat frequency, and the phase difference is 0. At this point, the expression for the sinusoidal pulsation waveform becomes Equation (11). All control algorithms were implemented and tested under synchronous configuration to eliminate confounding effects from pumping phase variability, as justified by myocardial recovery-targeted hemodynamic requirements [16].

(10) $$Aopr(t)=Aop_{0}+Aop_{a}\cdot \sin(2\pi t/T+\phi )$$

(11) $$Aopr(t)=Aop_{0}+Aop_{a}\cdot \sin(HR\cdot \pi t/30)$$

#### 2.2.2. The FFC-Based Pulsatile Control Algorithm

A good FFC link can be predicted to get most excitations. The excitation due to feedback errors is then used to correct the FFC excitation and resist disturbances based on the current response state. Feed-forward and feedback control compensate and offload each other, thus improving the performance of the system.

The controlled object RBP-CVS is a time-varying sixth-order complex system, and its transfer function in the frequency domain is written as *G*(*s*). In a unit-negative feedback control system, the transfer function of the FFC link is usually chosen to be the inverse of the controlled object *G^−^*^1^(*s*). However, the system characteristics vary with time due to the presence of time-varying parameters. Meanwhile, calculating higher-order complex feedforward excitations also leads to a slower response, so in order to design low-order effective FFC transfer functions, the RBP-CVS coupled inverse model needs to be simplified appropriately.

In this study, the following assumptions are imposed on the coupled RBP-CVS to design the FFC link:

A1: Loss of active contractility of the left ventricle, with all blood flow via the RBP.

A2: Neglecting left atrial compliance, central venous pressure and left atrial pressure are considered to be the same and constant at 15 mmHg, noted as Pv.

A3: Mitral valve always open, aortic valve always closed.

**Figure 6:**
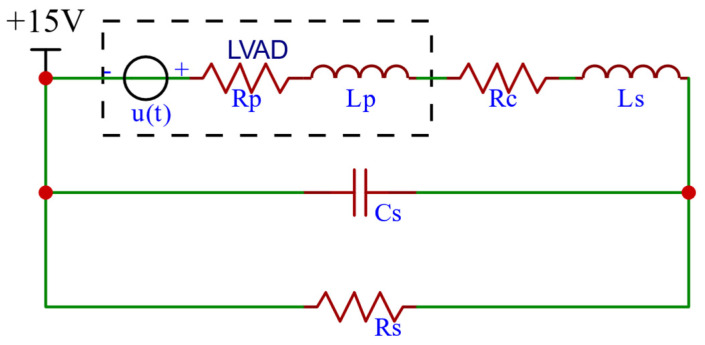
The equivalent circuit of RBP-CVS simplified by the assumptions.

Thus, the equivalent circuit of RBP-CVS can be simplified to the circuit shown in Figure 6:

In the FFC pathway, the input is a given aortic pressure signal *Aopr*(*t*), notated as *AOPr*(*s*) in the frequency domain, and the output is the voltage *β*·*ω*^2^ of a variable voltage source determined by the RBP speed, notated as *U*(*s*) in the frequency domain.

The total impedance of the simplified equivalent circuit in the frequency domain is given by Equation (12), and the total impedance of the simplified equivalent circuit with the RBP portion removed is given by Equation (13).

(12) $$Z_{1}(s)=(Lp+Ls)\cdot s+(Rp+Rc)+\frac{Rs\cdot \frac{1}{Cs\cdot s}}{Rs+\frac{1}{Cs\cdot s}}$$

(13) $$Z_{2}=Ls\cdot s+Rc+\frac{Rs\cdot \frac{1}{Cs\cdot s}}{Rs+\frac{1}{Cs\cdot s}}$$

**Figure 7:**
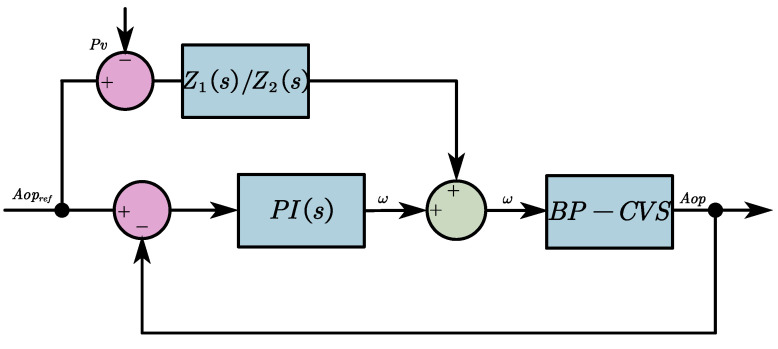
Schematic diagram of the system structure under the action of the FFC-based pulsatile controller.

The structure of the system under the action of the FFC-based pulsatile controller is shown in Figure 7 combined with the input-output relationship of the FFC path according to Kirchhoff’s circuit law and the expressions are as shown in Equation (14):

(14) $$\frac{U(s)}{AO\mathrm{Pr}(s)-Pv(s)}=\frac{Z_{1}(s)}{Z_{2}(s)}$$

#### 2.2.3. The Anti-Reflux Algorithm

In order to avoid the occurrence of the return flow phenomenon at certain low speed moments, this paper utilizes the pump flow signal *Qp* obtained from physiological feedback as a return flow discriminator. This signal is detected on the principle that there is a correspondence between rotational speed, current, and flow rate when the motor is rotating [8], so no additional flow sensor is needed.

**Figure 8:**
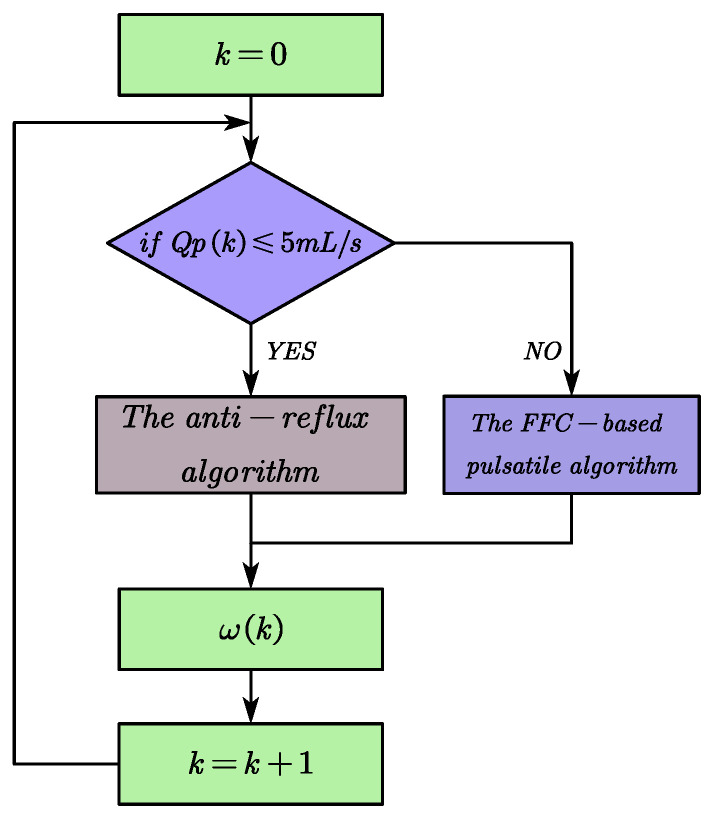
Schematic diagram of the switching process of pulsatile physiological control algorithm.

Considering the effect of the RBP flow estimation error and maximizing the enhancement of aortic pulsatility, in this paper, the critical value of regurgitation detection is set to 5 mL/s, at which point the RBP is considered to be operating at the zero point, where there is exactly no regurgitation. When the pump flow signal *Qp* is lower than the critical value of 5 mL/s, it is considered that there is a risk of reflux, and the algorithm is enabled, and the rotational speed is increased at a rate of 35 r/s to prevent further occurrence of the reflux phenomenon. When the anti-reflow algorithm is activated, the pulsation controller is dormant. When the flow signal *Qp* ≥ 5 mL/s, the pulsation controller will act, thus enhancing the pulse pressure. The speed update strategy is shown in Equation (15). Combining the FFC-based pulsatile control algorithm and the anti-reflux algorithm is called the pulsatile physiological control algorithm, and the switching process is depicted in Figure 8

(15) $$\omega (k+1)=\omega (k)+\Delta \omega ,\left\{\begin{array}{l} \Delta \omega =35,Qp\leq 5mL/s\\ \Delta \omega =0,Qp>5mL/s \end{array}\right.$$

## 3. Results and Discussion

### 3.1. Numerical Simulation Validation

#### 3.1.1. Tracking Performance Evaluation

**Figure 9:**
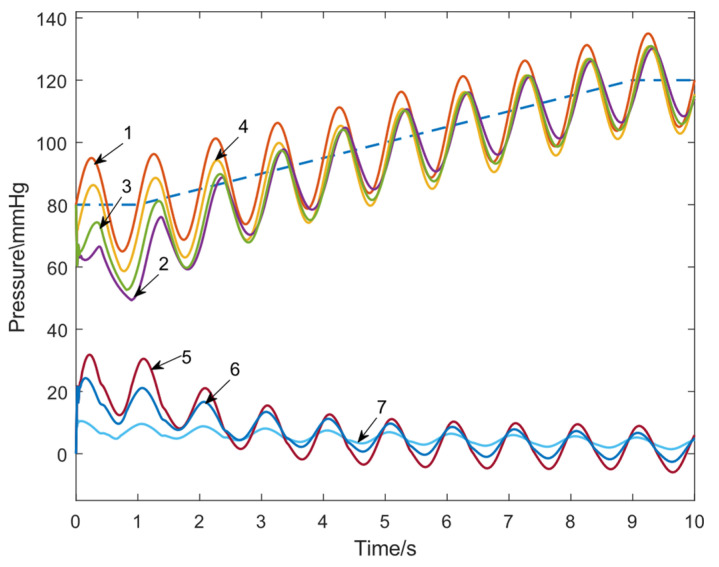
Response and tracking error signal curves of the system without FFC. 1. Reference Signal Input; 2. Response of the system without FFC at *Kp *= 50; 3. Response of the system without FFC at *Kp *= 100; 4. Response of the system without FFC at *Kp *= 300; 5. Tracking Error of the system without FFC at *Kp *= 50; 6. Tracking Error of the system without FFC at *Kp *= 100; 7. Tracking Error of the system without FFC at *Kp *= 300. The blue dotted line indicates the value of *Aop*_0_.

In order to compare the performance difference between the pulsatile physiological control (referred to as the system with FFC) and the no FFC pulsation control system (referred to as the system without FFC), a SIMULINK simulation test was conducted. The PI feedback control part of the integral gain *Ki *= 50; the proportional gains *Kp* are set to 50, 100, and 300, respectively; and the reference amplitude *Aop*_a_ = 50. In the time range of 1~9 s, the reference base value *Aop*_0_ is 80~120 mmHg, and the slope is 5 mmHg/s. The response curve of the system without the FFC link is shown in the following figure. The response curve of the system without the FFC link is shown in Figure 9. From Figure 9, it can be seen that under the condition of *Kp *= 300, the trough tracking error of one cycle increases instead, indicating that at this time it is no longer possible to improve the system response by increasing *Kp.* In addition, the overall response is still slow, and there exists an average tracking error of 5.187 mmHg, which cannot meet the fast response requirement.

**Figure 10:**
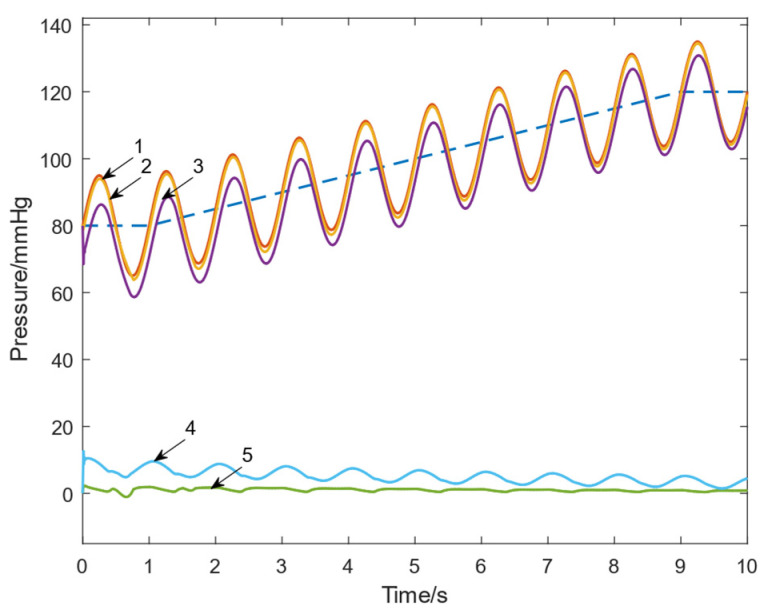
Tracking performance at *Kp *= 300. 1. Reference Signal Input; 2. Response of the system with FFC; 3. Response of the system without FFC; 4. Tracking Error of the system without FFC; 5. Tracking Error of the system with FFC. The blue dotted line indicates the value of *Aop_0_*.

Figure 10 illustrates the reference tracking performance of the systems with and without the FFC loop under the conditions of *Kp *= 300 and *Ki *= 50. The aortic pressure response of the system with FFC nearly overlaps with the desired signal line, exhibiting almost no steady-state error. Table 3 compares the tracking errors of the system for different proportional gains (*Kp *= 50, 100, 300). After introducing feedforward compensation, the maximum error reduction ratio ranges from 80% to 82%, and the average error reduction ratio ranges from 79% to 80%. For instance, when *Kp *= 300, the maximum error decreases from 35.17 mmHg to 6.833 mmHg, and the average error decreases from 6.920 mmHg to 1.437 mmHg. As *Kp* increases, the error further diminishes, demonstrating that the FFC loop effectively enhances system response accuracy and provides stable optimization across different control parameters. Moreover, the system response error shows negligible variation with changes in *Kp*, indicating that the system with FFC is insensitive to proportional coefficient adjustments. This significantly reduces the difficulty of parameter tuning in practical applications.

#### 3.1.2. Auxiliary Performance Evaluation

**Figure 11:**
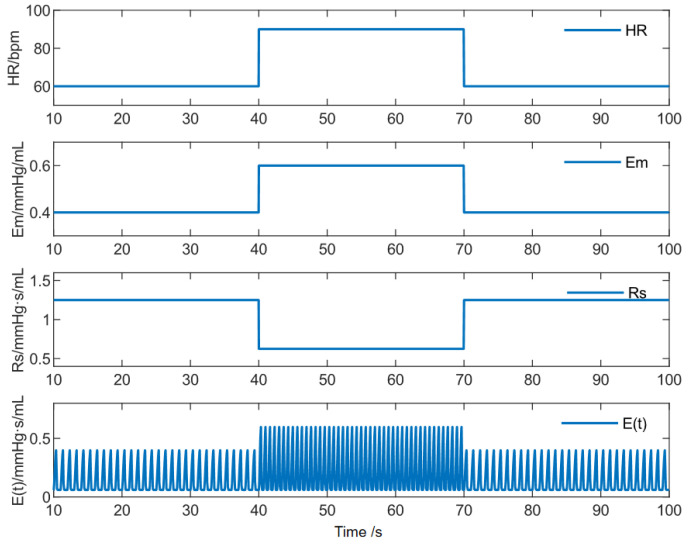
The variation of physiological parameters.

The metabolic levels of the human body vary between rest and activity, leading to corresponding changes in the cardiovascular system (CVS). When transitioning from rest to exercise, the body’s self-regulation mechanisms adjust cardiovascular resistance, enhance sympathetic nerve activity, and manifest as increased heart rate (HR), improved myocardial contractility, and reduced cardiovascular afterload, thereby boosting blood output. In this study, changes in patient activity were simulated by simultaneous step changes in HR, left ventricular maximum elastance (Emax), and systemic vascular resistance (Rs). If the controller can withstand step disturbances more severe than those occurring in the human body, its robustness can be considered reliable. By increasing/decreasing the baseline values of HR, Emax, and Rs by 50% (Figure 11 and Table 4), the transition from rest to exercise was simulated. Real-time monitoring of the electrocardiogram ensured that the target aortic pressure frequency matched the real-time HR for synchronized pulsation. The baseline aortic pressure *Aop*_0_ was set to 100 mmHg to meet blood demand during normal activity, while the amplitude *Aop*_a_ was set to 15 mmHg to achieve a pulse pressure of 30 mmHg. All simulations lasted 100 s, with activity state transitions (“rest-exercise-rest”) occurring at 40 and 70 s.

**Figure 12:**
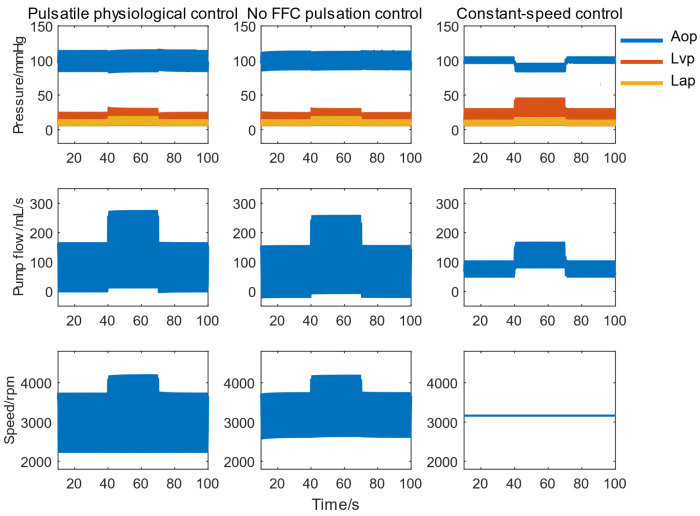
Systemic response of three control methods to a 50% variation in physiological parameters.

Figure 12 shows the responses of pulsatile physiological control, no FFC pulsation control, and constant-speed control to abrupt changes in activity intensity. All three methods provided a perfusion rate of 4.3 L/min at rest. The pulsatile physiological control maintained a minimum pump flow above 0 mL/s. During exercise, it delivered a peak blood flow of 276 mL/s, representing an increase of 19 mL/s compared to pulsatile control without a feedforward loop and 111 mL/s compared to constant-speed control. While pulsatile control without a feedforward loop maintained pulsatile aortic pressure and blood flow, it incurred pump reflux. Constant-speed control avoided reflux but generated only about 8 mmHg pulse pressure, with a 9 mmHg drop in mean arterial pressure during exercise. No abnormal changes in other blood variables or ventricular suction were observed.

**Figure 13:**
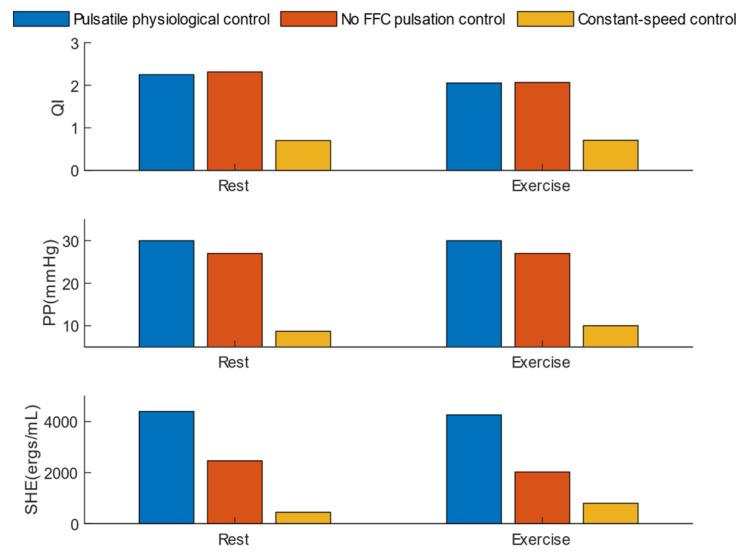
Comparison of hemodynamic pulsatility performance among three control strategies.

Figure 13 illustrates three pulsatility metrics under rest and exercise conditions: the pulsatility index (QI), pulse pressure (PP), and surplus hemodynamic energy (SHE). In both rest and exercise scenarios, the pulsatile physiological control consistently provided the highest and most stable PP and SHE, significantly outperforming constant-speed control. Specifically, the pulsatile physiological control achieved an SHE of 4396 ergs/mL, which is twice that of the no FFC pulsation control and 5–10 times that of constant-speed control. Although the QI provided by the pulsatile physiological control was slightly lower than that of the no FFC pulsation control during rest, this indicates that the pulsatile physiological control effectively activates the anti-reflux mode during low-speed operation.

**Figure 14:**
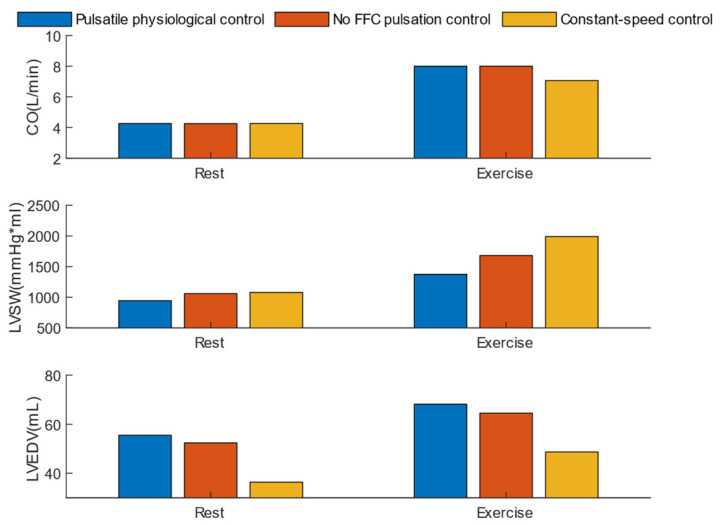
Comparison of ventricular unloading performance among three control strategies.

Figure 14 demonstrates the left ventricular unloading metrics under three control methods: total cardiac output (CO), left ventricular stroke work (LVSW), and left ventricular volume difference (LVVD). During the transition from “rest” to “exercise”, the blood demand increases, and both pulsatile physiological control and non-FFC pulsation control provide approximately 8 L/min of perfusion. In both rest and exercise scenarios, the pulsatile physiological control achieves the largest LVVD while maintaining the lowest LVSW.

### 3.2. In Vitro Experimental Validation

**Figure 15:**
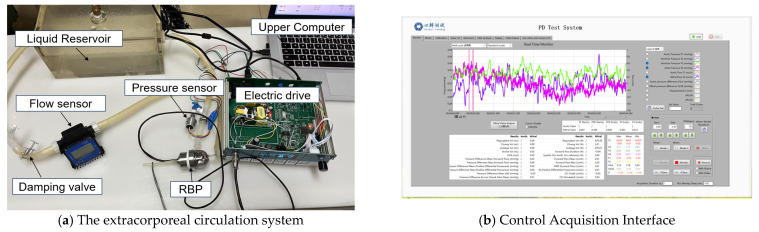
Schematic diagram of extracorporeal circulation device.

The experiment in this paper evaluates the feasibility of the designed regulator in practical use through a simple extracorporeal circulation device. The experimental bench consists of a storage tank, a damping valve, a circulation line, a centrifugal blood pump and its driver, a pressure sensor (range 0–120 KPa, accuracy 0.5%, supply voltage 12–36 V, output voltage 4–20 mV) and a flow indicator meter (range 0.1–10 L/min accuracy 1%). As shown in Figure 15, the pressure sensor measures the outlet pressure of the blood pump, which is considered the aortic pressure in the experiment. The pump outlet pressure signal is input to the upper computer through the pressure acquisition program of the PCB drive system, while the signal can be displayed in real time in the LabVIEW2017 software interface, the operation interface is shown in Figure 15 where the variable to be measured is aortic pressure P1 (red line), and the acquired pressure signal is stored in real time in the mainframe of the upper computer. The FFC regulator is implemented in the software LabVIEW, and its output accurately changes the rotational speed of the blood pump through the blood pump electric drive. In addition, the resistance of the circulating line can be adjusted by a damping valve [17].

**Figure 16:**
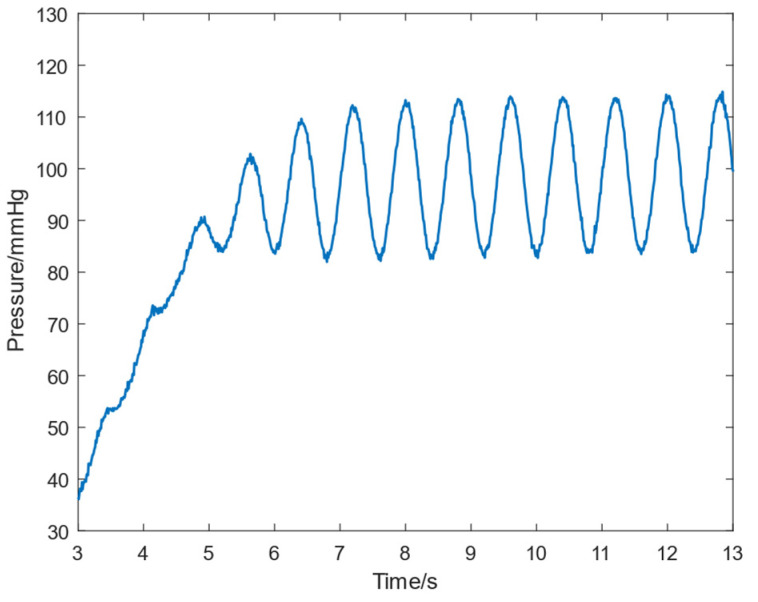
Aortic pressure signaling in in vitro experiments.

Figure 16 presents the in vitro experimental results of the physiological controller. Consistent with numerical simulations, the controller effectively reached the desired setpoint. After stabilization, the RBP outlet pressure increased to 83.5–114.8 mmHg (aortic pressure in the figure), generating a pulse pressure of approximately 30 mmHg. Simultaneously, a systemic flow rate of about 4.5 L/min was maintained [18].

### 3.3. Discussion

Existing studies have confirmed that reduced vascular pulsatility under CF-RBP support is a significant cause of vascular endothelial and smooth muscle cell dysfunction [15]. Most published literature focuses on providing physiological perfusion and anomaly detection for various types of RBPs. The proposed algorithm enhances pulsatility to near-physiological levels while ensuring physiological perfusion and preventing reflux.

In studies applying feedforward control loops, Zhao et al. [19] utilized a nonlinear model predictive control (NMPC) algorithm to regulate the coupling model of blood pumps and the cardiovascular system. NMPC, as a model-based feedforward control technique, enhances the control accuracy and stability of the RBP-CVS coupling model. However, due to computational resource limitations, this research did not validate the practical performance of NMPC. In contrast, the feedforward loop proposed in our study is simpler and more amenable to engineering implementation.

By constructing a feedforward-feedback composite control structure based on CVS characteristics, the proposed algorithm significantly improves aortic pressure tracking accuracy. In experiments simulating “rest-exercise-rest” transitions, the pulsatile physiological control demonstrated rapid and stable responses even under a 50% variation in model parameters, showcasing strong adaptability. In contrast, while no FFC pulsation control maintained pulsatile aortic pressure and blood flow, it suffered from pump reflux issues. In contrast, pulsation physiological control mitigates backflow by gradually reducing the speed of the pump when it reaches a flow rate below a critical point. Constant-speed control, on the other hand, was inadequate in providing pulsatility and adapting to changes in blood demand.

To quantitatively evaluate the effectiveness of different control algorithms, this study selected metrics for pulsatility performance and ventricular unloading. In terms of pulsatility, the pulsatile physiological control delivered optimal PP and SHE values. Additionally, healthcare professionals can conveniently customize the desired pulse pressure by adjusting AOPa, a result also validated in in vitro experiments. Regarding ventricular unloading, the pulsatile physiological control provided approximately 8 L/min of perfusion during exercise, consistent with the findings of Meki et al. [20], indicating that pump flow can automatically adjust to match perfusion needs. It has been suggested that reduced end-systolic and end-diastolic ventricular volumes may lead to myocardial atrophy and degeneration [21]. Asynchronous pump speed regulation results in a wider range of ventricular volume changes, potentially reducing myocardial atrophy and promoting recovery. However, Bozkurt [22] argues that asynchronous regulation generates non-physiological pressures and flows, which may be detrimental to long-term treatment. The designed pulsatile physiological control synchronizes the desired signal with the ECG, achieving the largest LVVD while maintaining the lowest LVSW, thus maximizing ventricular unloading and promoting myocardial cell recovery.

Although significant progress was made in blood flow pulsation regulation in this study, whether the pulsation pattern affects blood compatibility also needs to be further explored [23,24], such as the prediction and assessment of hemolysis based on the simulation of the 3D flow field of the blood pump. In addition, as VADs are implantable devices, the efficiency of the motor directly affects the battery life and the heating of the device [25,26,27]. Excessive temperature rise may lead to surrounding tissue damage or affect the stability of the motor and control system. Future research could further optimize the motor driving algorithm and adopt control strategies with better energy efficiency.

## 4. Conclusions

This study designed a pulsatile physiological control system to enhance the pulsatility and physiological performance of rotary RBPs. First, an RBP-CVS simulation model was established in Simulink. Second, an FFC loop based on CVS characteristics was designed, significantly improving the tracking accuracy of aortic pressure under pulsatile reference waveforms. Additionally, an anti-reflux control algorithm was developed to prevent reflux events during low-speed operation.

The effectiveness of the pulsatile physiological control system was validated through numerical simulations, demonstrating the following results:

1. The designed FFC loop reduced the maximum tracking error by approximately 81% and the average error by about 80%.
2. The pulsatile physiological control achieved rapid and stable responses under high disturbances with a 50% variation in CVS parameters, effectively preventing reflux events.
3. The system provided excellent pressure and flow pulsatility, generating a physiological pulse pressure of 30 mmHg during both exercise and rest. The delivered SHE was 5–10 times that of constant-speed control.
4. The system achieved the largest LVVD while maintaining the lowest LVSW, promoting ventricular unloading and myocardial recovery.

Furthermore, in vitro experiments demonstrated an increase in aortic pressure to 83.5–114.8 mmHg, producing a physiological pulse pressure of 30 mmHg. These results confirm that the designed controller is suitable for practical RBP motor control applications.

## Figures and Tables

**Table 1:** Characteristic parameter values of the CVS system [13].

Parameter	Value	Unit	Meaning
*HR*	$HR_{base}$	bpm	Heart rate
$E_{max}$	$E_{maxbase}$	mmHg/mL	Left ventricular end-systolic compliance
$E_{\min}$	0.0600	mmHg/mL	Left ventricular end-diastolic compliance
*Rs*	$Rs_{base}$	mmHg·s/mL	Peripheral vascular resistance
*Rm*	0.0050	mmHg·s/mL	Mitral valve resistance
*Ra*	0.0010	mmHg·s/mL	Aortic valve resistance
*Rc*	0.0398	mmHg·s/mL	Aortic resistance
*Cr*	4.4000	mL/mmHg	Left atrial compliance
*Ca*	0.0800	mL/mmHg	Aortic compliance
*Cs*	1.3300	mL/mmHg	Peripheral vascular compliance
$C\left(t\right)$	$1/E\left(t\right)$	mL/mmHg	Left ventricular compliance
*Ls*	0.0005	mmHg·s^2^/mL	Aortic blood inertia
*Dm*	-	-	Mitral valve
*Da*	-	-	Aortic valve
*Rp*	−0.00183	mmHg·s^2^/mL	Blood pump and cannula equivalent inertia
*Lp*	−0.39100	mmHg·s/mL	Blood pump and cannula equivalent resistance
β	1.147 × 10^−5^	mmHg·s^2^/rad^2^	Blood pump speed related constants

**Table 2:** Initial values of system state variables [13].

Scheme	Sign	Initial Value	Meaning
$x_{1}$	*Vlv*(*t*)	140 mL	Left ventricular volume
$x_{2}$	*Lap*(*t*)	7.6 mmHg	Left atrial pressure
$x_{3}$	*Ap*(*t*)	67 mmHg	Arterial pressure
$x_{4}$	*Aop*(*t*)	80 mmHg	Aortic pressure
$x_{5}$	*Q*(*t*)	0 mL/s	Aortic flow
$x_{6}$	*Qp*(*t*)	0 mL/s	Pump flow

**Table 3:** Comparison of Response Errors with and without FFC for different proportional gains.

Proportional Gain	With or Without FFC	Maximum Error (mmHg)	Reduction Ratio	Average Error (mmHg)	Reduction Ratio
Kp=50	without	35.17	81%	6.920	79%
	with	6.833		1.437	
Kp=100	without	24.45	80%	6.715	80%
	with	4.911		1.376	
Kp=300	without	12.93	82%	5.187	80%
	with	2.325		1.035	

**Table 4:** CVS model parameter values after ±50% Variation.

Parameter	Baseline Value	Resting State	Exercise State
*HR*	60 bpm	60 bpm	90 bpm
*Emax*	0.4 mmHg/mL	0.4 mmHg/mL	0.6 mmHg/mL
*Rs*	1.25 mmHg·s/mL	1.25 mmHg·s/mL	0.75 mmHg·s/mL

## Data Availability

Data available on request. The data underlying this article will be shared on request to the corresponding author.

## Abbreviations

The following abbreviations are used in this manuscript:

RBP	Rotary Blood Pump
FFC	Feed-forward compensation
CVS	Cardiovascular system
ECG	Electrocardiogram
LVAD	Left Ventricular Assist Device
HR	Heart rate
Emax	Maximum elastance
Rs	Resistance
QI	Pulsatility index
PP	Pulse Pressure
SHE	Surplus hemodynamic energy
CO	Total cardiac output
LVSW	Left ventricular stroke work
LVVD	Left ventricular volume difference
NMPC	Nonlinear model predictive control
